# Low-Temperature Fibre Direct Compounding of Cellulose Fibres into PA6

**DOI:** 10.3390/ma15196600

**Published:** 2022-09-23

**Authors:** Janez Slapnik, Yuanxi Liu, Robert Kupfer, Thomas Lucyshyn, Blaž Nardin, Gerald Pinter

**Affiliations:** 1 Faculty of Polymer Technology, Ozare 19, 2380 Slovenj Gradec, Slovenia; 2 Institut Fuer Leichtbau und Kunststofftechnik, Technische Universitaet Dresden, Holbeinstrasse 3, 01307 Dresden, Germany; 3 Montanuniversitaet Leoben, Otto Gloeckel-Strasse 2, 8700 Leoben, Austria

**Keywords:** injection moulding, low-temperature processing, fibre direct compounding, thermoplastic matrix composites, engineering polymers, renewable fibres, polyamide, cellulose fibres

## Abstract

This study reports on the development of a novel polymer processing approach that combines low-temperature (LT) processing and fibre direct compounding (FDC) to reduce the thermal stress on thermosensitive components that occurs during compounding and subsequent injection moulding (IM). Composites based on polyamide 6 (PA6) and cellulose fibres (CeF) were prepared using an LT-FDC process and in parallel with a conventional approach using a twin-screw extruder and IM. The morphological, optical, thermal, and mechanical properties of the prepared samples were investigated using optical microscopy (OM), differential scanning calorimetry (DSC), colorimetry, dynamic mechanical analysis (DMA) and tensile tests. Composites prepared using LT-FDC exhibited worse fibre dispersion but lower fibre degradation. In comparison to neat PA6, the LT-FDC composites had increased tensile modulus (*E*_t_) and storage modulus (*E′*) at 120 °C by up to 32% and 50%, respectively, while the tensile strength (*σ*_m_) decreased by 20%.

## 1. Introduction

Thermoplastic matrix composites (TMC) reinforced with renewable fibres (RF) are an interesting class of structural materials due to several advantages over conventional TMC reinforced with non-renewable fibres (e.g., glass fibres), such as reduced depletion of non-renewable resources, lower carbon dioxide (CO_2_) footprint, lower density, and lower abrasion on processing equipment. Lignocellulosic fibres (LCeF) and CeF are particularly attractive reinforcements for TMC due to their large abundance, high specific mechanical properties, and higher thermal stability compared to other RF. However, there are many challenges and limitations related to LCeF- and CeF-reinforced TMC, such as poor fibre–matrix interactions, high moisture uptake and relatively low thermal stability of the fibres. The latter limitation has led most efforts to focus on the development of TMC based on polymers with a low glass transition (*T*_g_) and low melting temperatures (*T*_m_), such as polyethylene (PE), polypropylene (PP), and polylactic acid (PLA). TMC reinforced with RF are especially attractive for the automotive sector due to requirements for environmental friendliness, lightweight materials with low cost. Mainly, they are used in less demanding interior applications because of their moisture sensitivity and relatively poor mechanical properties [1,2,3,4]. RF-reinforced TMC based on engineering polymers promise to offer superior mechanical and thermal properties, thus expanding their potential range of applications [5,6,7,8,9]. However, the processing of TMC reinforced with LCeF and CeF at temperatures above 220 °C leads to a significant deterioration of the mechanical properties of the composites accompanied by colour changes [10]. This limiting temperature is well below the typical processing temperatures of engineering thermoplastics, such as PA6 [5,6] polyamide 6.10 [6], polybutylene terephthalate (PBT) [7], polytrimethylene terephthalate (PTT) [7], and polyethylene furanoate (PEF) [8], which are usually processed above 240 °C. Therefore, for the successful preparation of engineering polymers reinforced with LCeF or CeF, it is important to reduce the thermal stress on the fibres during processing as much as possible [9]. Polyamides are attractive matrices for CeF-reinforced TMC due to their excellent mechanical and thermal properties and favourable matrix–fibre interactions resulting from hydrogen bonding. Moreover, they can be produced from renewable resources, thus offering the possibility of producing fully renewable composites [9,11]. However, besides high processing temperatures, they also have their limitations, such as high moisture absorption and associated plasticisation, leading to a decrease in *T*_g_ with a concomitant decrease in modulus and strength, and their tendency for chemical degradation (hydrolysis and oxidation), leading to embrittlement [12,13,14]. One of the first attempts to prepare composites based on PA6 and CeF was made by Sears et al., who introduced two innovative approaches. First, they used fibre pelletizing techniques to reduce the bulk density of CeF, thus eliminating challenges that usually occur with the dosing of low bulk density materials. Second, they introduced the low-temperature processing (LTP) approach, in which fibres are dosed into the extruder using a side feeder, while extruder barrel temperatures after the side feeding are reduced below the melting point of the matrix material, and the fibres are compounded into a supercooled melt. The LTP approach resulted in significantly lower CeF degradation during the compounding process. However, after compounding, the composites were processed by IM, where they were still exposed to temperatures above the melting point of the matrix [15]. Since then, many new innovative approaches have been developed to produce polyamides reinforced with LCeF and CeF, which can be divided into three main categories: process modification, fibre modification, and matrix modification [9]. Injection moulding compounding (IMC) is a processing technique that was first introduced by Krauss Maffei (KM) in 1998. IMC combines compounding and IM into one step, thus in theory offering significant benefits in terms of decreased thermal and shear stress on the processed material and lower energy consumption. KM IMC technology uses a twin-screw extruder connected to the IM machine via a melt pump and melt accumulator, which enables the joining of a continuous compounding process with a discontinuous IM process [16,17]. Since then, similar technologies that combine compounding and IM into one step have been introduced by other manufacturers [18,19]. One of the newer technologies is Arburg’s FDC, in which a continuous roving of, usually, glass fibres is cut and fed directly into an IM machine’s barrel via a side feeder [20]. FDC has the potential to further reduce the thermal stress on the processed material and energy consumption compared to classical IMC. However, unmodified FDC is not well suited for processing CeF due to challenges that occur with the cutting and dosing of tough and flexible CeF. Although these one-step technologies promise to reduce the thermal stress on the processed materials, which is one of the main challenges in the preparation of RF-reinforced TMC based on engineering polymers, their application in this field is completely unexplored in the scientific literature.

The aim of the present study was to modify FDC for the processing of CeF and to evaluate its advantages and limitations for the preparation of engineering polymers reinforced with CeF. In addition, the study aimed to combine the LTP approach with FDC to further reduce thermal stress during processing. In this way, CeF could be processed only in the supercooled melt, which should result in a significantly lower thermal load on CeF, compared to a two-step process or conventional FDC. Decreased thermal load on the fibres should be reflected in the colour of the composites, as CeF undergoes colour changes upon exposure to high temperatures.

## 2. Materials and Methods

### 2.1. Materials

PA6 Akulon^®^ K222-D (DSM Engineering Materials, Emmen, The Netherlands) was used as the matrix material. According to the manufacturer, the PA6 had a melt volume–flow rate (MVR) of 185 cm^3^/10 min (ISO 1133, 275 °C, 5 kg), a melting point of 220 °C (ISO 11357-3, 10 K/min), and a density of 1.13 g/cm^3^ (ISO 1183). Lyocell fibres Tencel^TM^ FCP-9 300 (Lenzing, Lenzing, Austria) were kindly provided by Lenzing. The lyocell fibres had a linear density of 1.7 dtex and a length of 300 µm. Erucamide Crodamide ER (Croda, Snaith, UK) was used as a slip agent.

### 2.2. Sample Preparation

#### 2.2.1. Pelletising

The fibres were pelletised using a PTA 50 (Tecno Aspira, Novedrate, Italy) pelletiser. The pelletiser had a 4 kW electric motor and a 6 mm die plate. Prior to pelletising, the fibres were conditioned to a moisture content of 20 wt.% and dry mixed with a slip agent (2 wt.% on dry mass) to reduce friction during the pelletising process and to enhance fibre dispersion in the polymer matrix.

#### 2.2.2. Compounding

Composites were prepared by melt-blending 20 wt.% of CeF pellets (CeFP) into PA6 using an LTE 20-44 (Labtech Engineering, Samut Prakan, Thailand) co-rotating twin-screw extruder. The extruder had a screw diameter of 20 mm and an L:D ratio of 44:1. Prior to compounding, cellulose pellets were dried in a laboratory oven 100–800 (Memmert, Büchenbach, Germany) at 105 °C, below a moisture content of 0.2 wt.%. A flat barrel temperature profile of 230 °C was used. The screw speed was 200 min^−1^.

#### 2.2.3. Injection Moulding and Fibre Direct Compounding

**Figure 1:**
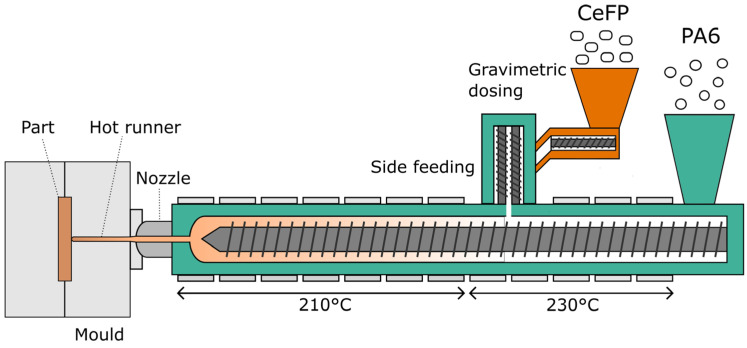
Schematic representation of low-temperature fibre direct compounding of cellulose fibre pellets into PA6.

The test specimens were produced with an Allrounder 920 S 4600 (Arburg, Lossburg, Germany) injection moulding machine, equipped with an FDC system. The machine had a clamping force of 5000 kN, an L:D ratio of 22.5, and a screw diameter of 80 mm. The mould had a hot runner system and a cavity volume of 388 cm^3^. The produced parts consisted of a flat plate and three specimens for tensile tests according to the ISO 527-2 standard (type 1A). The composites were dried in the dry air dryer at 105 °C, below a moisture content of 0.1 wt.%. The standard processing parameters are summarised in Table 1. Two different barrel temperatures were used: a flat temperature profile and an LT profile. For FDC, a rowing–cutting system was replaced by a MINICOLOR G (motan-colortronic, Isny im Allgaeu, Germany) gravimetric dosing system to enable the dosing of CeFP. The schematic representation of the LT-FDC process is presented in Figure 1. The dosing of CeFP was calibrated by weighing the injection moulded parts produced from composites, calculating the required mass of the pellets, and setting the required mass flow rate of the gravimetric dosing system. The calibration was validated by weighing the mass of the produced parts and comparing it to the mass of composite parts prepared by a conventional two-step process.

Eight different samples were prepared, which differed in terms of material composition, processing techniques and processing parameters (Table 2). The reference sample (PA) was neat PA6 processed by a conventional IM process. The sample PA_LT was neat PA6 processed using an LT approach to investigate the influence of the LT profile on the properties of the matrix. The samples PA-C and PA-C_LT were composites produced by a two-step process using a conventional and LT-IM technique, respectively. Samples PA-C_FDC and PA-C_FDC_LT1 were composites produced using an FDC technique using a flat and LT profile, respectively. Samples PA-C_FDC_LT2 and PA-C_FDC_LT3 were composites produced using the LT-FDC technique, but with different processing parameters, with the aim to enhance the fibre dispersion. Before characterisation, the samples were conditioned according to ISO 291 standard (at least 88 h at 50 ± 10% of relative humidity and 23 ± 2 °C).

### 2.3. Characterisation

#### 2.3.1. Thermogravimetric Analysis

The decomposition behaviour of fibres and PA6 granules was investigated using a TGA/DSC 3+ (Mettler Toledo, Greifensee, Switzerland) simultaneous TGA/DSC instrument in 40 µL aluminium crucibles. Non-isothermal measurements were performed by heating the samples from 40 °C to 550 °C, with a heating rate of 10 K/min in a nitrogen (N_2_) atmosphere (20 mL/min). Isothermal measurements were performed by heating the samples from 40 °C to the defined temperature with a heating rate of 50 K/min in the N_2_ atmosphere (20 mL/min), followed by a 20 min isothermal segment in the N_2_ or oxygen (O_2_) atmosphere (20 mL/min).

#### 2.3.2. Optical Microscopy

Optical micrographs were captured using a VHX-7000 (Keyence, Osaka, Japan) digital microscope using a VH-ZST lens. Micrographs of the fracture surfaces of the tensile tested specimens were captured using ring illumination at 20× magnification. Fibre dispersion and distribution were evaluated by preparing approximately 20 µm thick slices of cross-sections of untested tensile test specimens using a microtome and capturing micrographs using a transmitted illumination at 300× and 500× magnification.

#### 2.3.3. Differential Scanning Calorimetry

Thermal properties were determined using a DSC 2 (Mettler Toledo, Greifensee, Switzerland) calorimeter in 40 µL aluminium crucibles. Specimens were prepared from tensile test specimens and had a mass of 3.5–5.0 mg. The samples were tested in the temperature range of 25 °C to 240 °C, with a heating/cooling rate of 10 K/min in the N_2_ atmosphere (20 mL/min). The isothermal segments before the heating and cooling segments were set to 1 min to minimise CeF degradation during the measurement. The results are average values of two measurements. The degree of crystallinity (*X*_c_) was calculated according to the following equation:

*X*_c_ = Δ*H*_m_/(*w*_PA_ × Δ*H*_0_) (1)

where Δ*H*_m_ is the sample melting enthalpy, *w*_PA_ is the mass fraction of PA6 in the sample, and Δ*H*_0_ is the melting enthalpy of 100% crystalline PA6 (230 J/g) [21].

#### 2.3.4. Colorimetry

The colour of samples was determined on tensile tests specimens using an NR60CP (Shenzhen ThreeNH Technology, Shenzhen, China) colorimeter. Measurements were performed based on the International Commission on Illumination (CIE) L*a*b* colour space using a D65 standard illuminate, a 4 mm aperture and a 10° observer angle. The results are average values of five measurements.

#### 2.3.5. Dynamic Mechanical Analysis

Dynamic mechanical properties were determined using a DMA 8000 (Perkin Elmer. Waltham, MA, USA) dynamic mechanical analyser according to ASTM D5418 standard. Specimens were prepared from tensile test specimens. Samples were tested in flexure using dual cantilever beam support. The distance between the supports was 12.6 mm, the frequency was 1 Hz, and the amplitude was 0.005 mm. Samples were tested from 26 °C to 160 °C, with a heating rate of 1 K/min.

#### 2.3.6. Tensile Test

Tensile properties were determined using an Ag-X plus 10 kN (Shimadzu, Kyoto, Japan) universal testing machine according to ISO 527-1 standard. The gauge length was 115 mm, preload was 10 N, and testing speed was 1 mm/min until 0.25% strain and 50 mm/min until the break. The results are the average values of five measurements.

## 3. Results

### 3.1. Decomposition Behaviour

**Figure 2:**
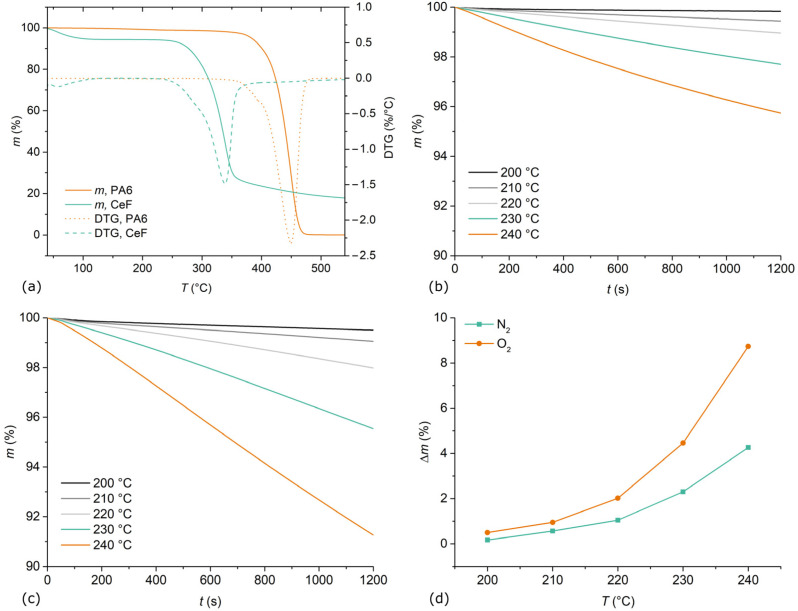
TGA results: (**a**) non-isothermal measurement of PA6 and CeF; (**b**) isothermal measurements of CeF in N_2_ atmosphere; (**c**) isothermal measurements of CeF in O_2_ atmosphere; (**d**) comparison of Δ*m* at 20 min for isothermal TGA measurements.

Non-isothermal TGA measurements were performed to evaluate the general decomposition behaviour of PA6 and CeF. In addition, isothermal TGA measurements of CeF were performed in the temperature range of 200 °C to 240 °C, which corresponds to the range to which CeF are exposed during processing. It was shown that isothermal TGA measurements of CeF in the N_2_ atmosphere at IM processing temperatures correlate well with the decomposition of the fibres during processing and, in turn, to the retention of their reinforcing effect [10]. TGA and derivative thermogravimetry (DTG) thermograms of non-isothermal TGA measurements of PA6 and CeF are presented in Figure 2a. PA6 exhibited a single mass loss (Δ*m*) step of 100%, with an initial decomposition temperature (IDT) of 420 °C, a decomposition temperature (*T*_d_) of 452 °C, and a decomposition rate (*R*_d_) of 0.23 min^−1^. CeF exhibited a two-step mass loss, with the first step (40–100 °C) corresponding to moisture evaporation (5%) and the second to the cellulose decomposition. CeF decomposed at significantly lower temperatures than PA6, with Δ*m* of 77%, IDT of 305 °C (−115 K), and *T*_d_ of 339 °C (−113 K). However, the *R*_d_ value of CeF was lower at 0.15 min^−1^. The thermograms of the isothermal TGA measurements in the N_2_ atmosphere are presented in Figure 2b. With increasing time, the *m* decreased more or less linearly, with a slight concave upward response that was especially pronounced at higher measurement temperatures. The thermograms of the isothermal TGA measurements in the O_2_ atmosphere are presented in Figure 2c. Similarly, as in the N_2_ atmosphere, CeF exhibited more or less linear Δ*m* with increasing time, but in this case with a slightly concave downward response. A comparison of Δ*m* at 20 min of isothermal TGA measurements in N_2_ and O_2_ atmospheres at different temperatures is presented in Figure 2d. In both atmospheres, Δ*m* increased exponentially with increasing temperature. In the N_2_ atmosphere, CeF were relatively stable up to 220 °C, while a significant increase in the decomposition rate was observed at 230 °C. In the O_2_ atmosphere, CeF decomposed to a higher extent compared to the N_2_ atmosphere at all investigated temperatures, with increasingly larger differences at temperatures above 220 °C. The results suggest that limiting both exposure time and temperature is essential to prevent the decomposition of CeF in a processing window of PA6. However, it appears that for practical applications, exposure temperature has a greater impact on CeF degradation (Δ*m*) because it exhibits an exponential response, while the relationship between the Δ*m* and exposure time is relatively linear.

### 3.2. Macroscopic Overview

**Figure 3:**
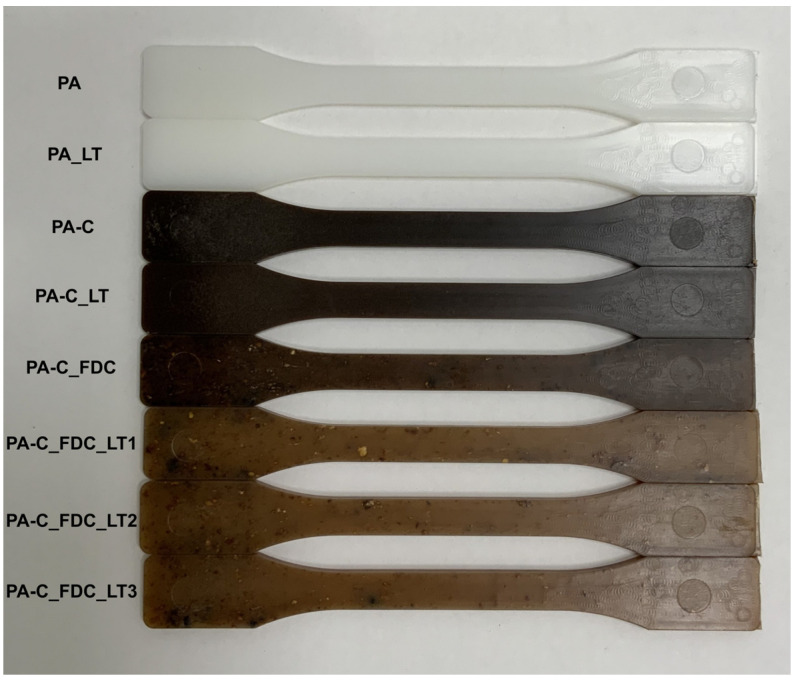
Macroscopic overview of the samples.

A macroscopic overview of the IM tensile test specimens of different samples is shown in Figure 3. No significant differences were observed between the samples PA and PA_LT. Sample PA-C was dark brown in colour and homogeneous, while sample PA-C_LT was similar but only slightly lighter in colour. The PA-C_FDC sample was significantly lighter in colour than the composites prepared by a two-step process but had a large number of visible agglomerates of non-dispersed CeF. The samples prepared by the LT-FDC process were light brown and had a large number of CeF agglomerates.

### 3.3. Microstructure

**Figure 4:**
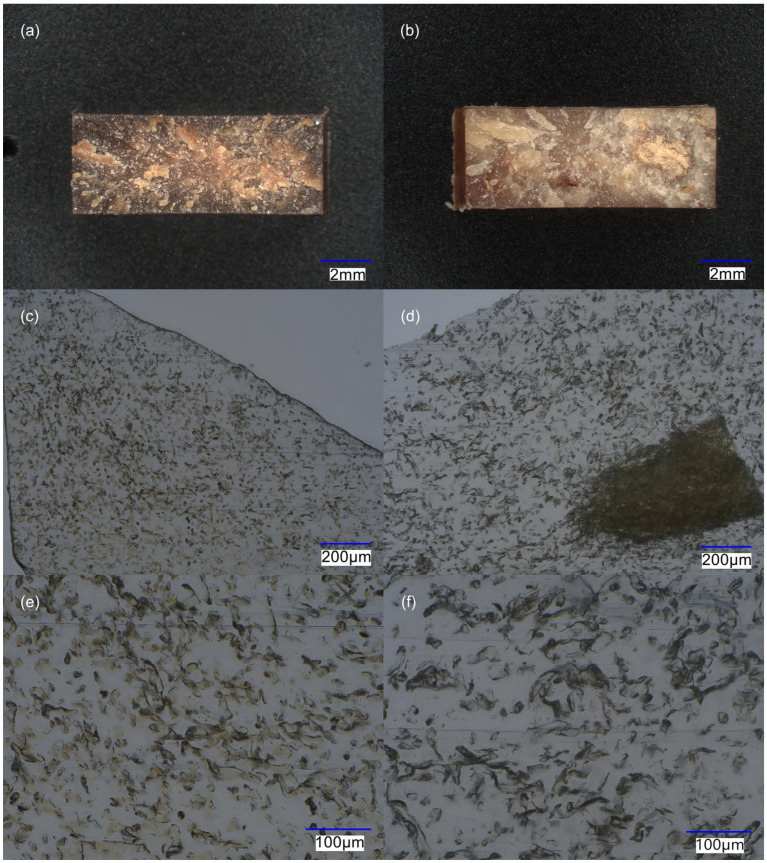
Optical micrographs of: (**a**) fracture surface of PA-C at 20× magnification; (**b**) fracture surface of PA-C_FDC_LT2 at 20× magnification; (**c**) microtome cut cross-section of PA-C at 300× magnification; (**d**) microtome cut cross-section of PA-C_FDC_LT2 at 300× magnification; (**e**) microtome cut cross-section of PA-C at 500× magnification; (**f**) microtome cut cross-section of PA-C_FDC_LT2 at 500× magnification.

Micrographs of fracture surfaces of the tensile tested specimens of samples PA-C and PA-C_FDC_LT2 are presented in Figure 4a,b, respectively. Samples prepared by a two-step process had similar microstructure that significantly differed from the samples prepared by a one-step process. At 20× magnification, the microstructure of the samples prepared by a two-step process was homogeneous and without visible agglomeration. In contrast, the samples prepared by a one-step process had visible agglomerates that differed in size, density, and colour. Some agglomerates looked like a network of entangled fibres that were not densely compressed and were up to about 2 mm in diameter, while other agglomerates appeared to be more compressed and were smaller. The agglomerates also differed significantly in colour. Some agglomerates were only slightly yellow, while others were dark brown. Micrographs of the microtome cut cross-sections of the untested specimens of samples PA-C and PA-C_FDC_LT2 are presented in Figure 4c,e and Figure 4d,f, respectively. The samples prepared by a two-step process had randomly distributed fibres that were well dispersed. In comparison, the samples prepared by a one-step process contained large agglomerates of non-dispersed fibres, while the majority of the fibres were randomly distributed and relatively well dispersed. In both samples, some fibres were oriented perpendicular to the flow direction, while a large portion of fibres were randomly oriented. The fibres in the samples prepared by a two-step process were light brown, while no visible colouration was observed in the fibres processed by the LT one-step approach. It is clear that the dispersion of the fibres is much better with the two-step processing. This is because a co-rotating twin-screw extruder is designed for excellent dispersion of particles/fibres, while FDC is specifically designed for processing long GF, where high mechanical stresses on the fibres are not desirable, as they would lead to shortening of the fibres and thus poorer mechanical properties. Compared to glass fibres, CeF have much lower stiffness (*E*_t_ of E-GF ≈ 70 GPa [1], *E*_t_ of lyocell ≈ 20 GPa [22,23]), resulting in fibre entanglement and lower flow-induced fibre orientation. Therefore, good mixing and high shear stresses are required for the efficient dispersion of CeF. Another important aspect is that in the present study, fibres were in the form of pellets that are much easier to dose but are known to result in poor fibre dispersion, even when processed with a twin-screw extruder [24,25,26]. The dispersion of CeF in the FDC process could therefore potentially be improved by using stiffer CeF, direct dosing of uncompressed fibres using a gravimetric dosing system capable of handling low-bulk density materials, or by using a specially designed screw geometry with good dispersive and distributive mixing capabilities.

### 3.4. Crystallisation and Melting Behaviour 

The thermal properties of samples determined by DSC are summarised in Table 3. Neat PA6 exhibited an onset crystallisation temperature (*T*_c, onset_) of 195.3 °C, while composites exhibited lower *T*_c, onset_ (0.8–1.4 K), depending on the processing technique. Composites processed in one step exhibited slightly higher *T*_c, onset_ compared to composites processed in two steps. A similar trend was found for crystallisation temperature (*T*_c_), where neat PA6 exhibited slightly higher *T*_c_ (0.9–2.1 K) compared to composites, and composites processed in two steps had slightly lower *T*_c_ compared to composites processed in one step. Lower *T*_c, onset_ and *T*_c_ of composites suggest that CeF does not promote heterogeneous nucleation, but rather sterically hinders the crystallisation of PA6 [27]. The *T*_m_ of neat PA6 was on average slightly higher compared to the composites, which was ascribed to the formation of smaller crystals in composites due to the crystallisation hindrance by the CeF. Samples of neat PA6 had higher values of crystallisation (Δ*H*_c_) and melting (Δ*H*_m_) enthalpy than composites due to the higher mass fraction of PA6. Estimated *X*_c_ of neat PA6 and composites processed in two steps was approximately in the same range, while composites processed in one step had slightly higher values of estimated *X*_c_, which can be explained by a lower content of dispersed CeF in the measured specimens, as a certain amount of CeF was in large agglomerates.

### 3.5. Colour

**Figure 5:**
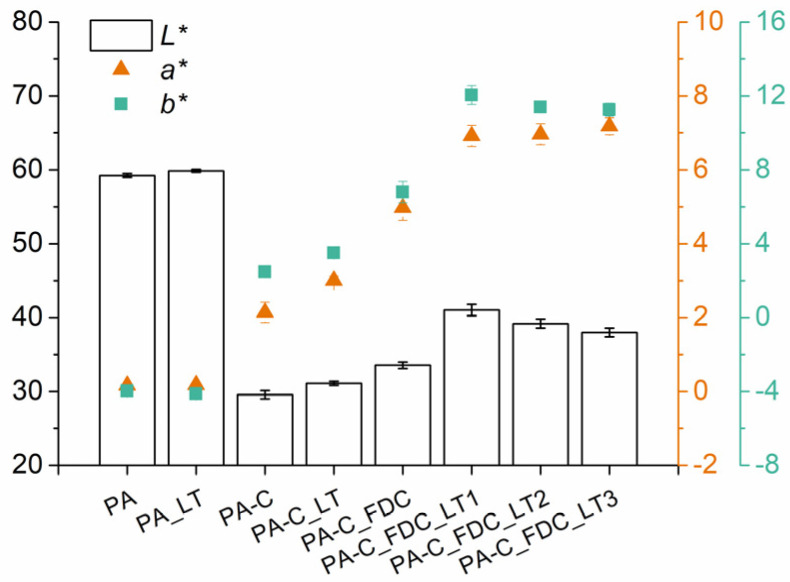
L*a*b* values of samples determined by colorimetry.

Colorimetry has been shown to be an important nondestructive characterisation technique for CeF and NF composites [10,28,29]. The colorimetry results are usually expressed in the CIE L*a*b* colour space, where the L* value represents perceptual lightness (0—black, 100—white), the a* value corresponds to the green–red opponent colours (negative values towards green, positive values towards red), and b* value corresponds to the blue–yellow opponent colours (negative values towards blue, positive values towards yellow). When lyocell fibres are exposed to high thermal stress during melt processing, colour changes occur that can be easily evaluated with colorimetric measurements. With increasing thermal stress, the composites become darker, while the a* and b* values first increase up to a certain point and then decrease. The colour of polypropylene composites reinforced with lyocell fibres (IM at high temperatures) has been shown to correlate well with the mechanical properties of composites [10]. Figure 5 presents the results of colorimetric measurements of the prepared samples. Neat PA6 processed using the LT-IM technique had a slightly higher L* value (+1%) than conventionally processed PA6, which can be attributed to lower thermo-oxidative degradation. Composites processed by a conventional two-step process had the lowest L*, a*, and b* values among the composites, which can be attributed to strong thermo-oxidative degradation of the fibres during HT processing. In comparison, composites processed by the LT-IM approach exhibited increased L* (+5%), a* (+41%), and b* (+41%) values, which was due to lower thermal stress. Composites processed in one step using a standard temperature profile exhibited significantly higher L* (+14%), a* (+132%), and b* (+174%) values compared to composites processed by a conventional two-step process. The processing of composites in one step using a LT profile further increased L* (+22%), a* (+39%), and b* (+77%) values compared to using a standard temperature profile, while higher *Q*_inj_, *ω*_screw_, and *P*_back_ resulted in a slight decrease of L* value due to additional shear heating. Results indicate that one-step processing significantly decreases CeF thermo-oxidative degradation in comparison to a conventional two-step processing, especially when using a LT approach.

### 3.6. Dynamic Mechanical Properties

**Figure 6:**
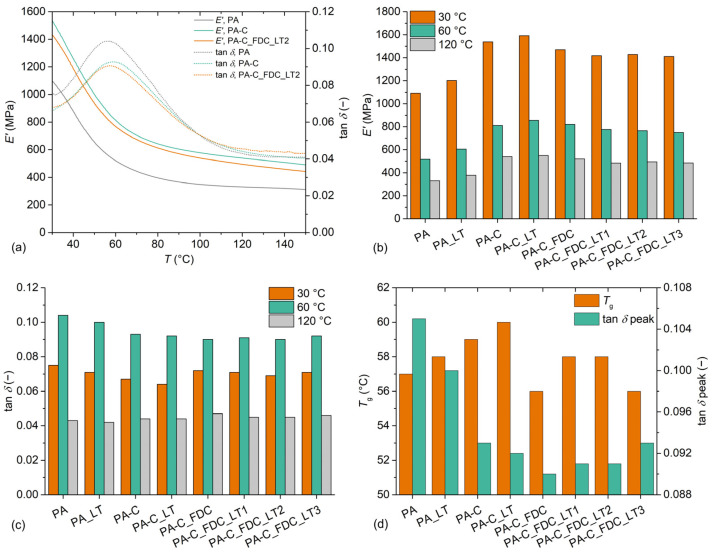
Dynamic mechanical properties of samples: (**a**) *E′* and tan *δ* as a function of the temperature of representative samples; (**b**) *E′* of samples at 30 °C, 60 °C and 120 °C; (**c**) tan *δ* of samples at 30 °C, 60 °C and 120 °C; (**d**) *T*_g_ and corresponding tan *δ* values of samples.

Figure 6 presents the dynamic mechanical properties of samples. *E′* and the loss factor (tan *δ*) as a function of temperature for the most representative samples are presented in Figure 6a. All samples exhibited relatively similar dynamic mechanical behaviour. In the region from 30 °C to 60 °C, the samples were in a leathery state, with a constant rate of decrease in *E′*. At about 60 °C, a peak in tan *δ* was observed, corresponding to *T*_g_. At this temperature, a transition from a leathery state to a rubbery plateau was observed. Compared to neat PA6, the composites exhibited a significantly higher *E′* over the entire studied temperature range, while tan *δ* was higher for neat PA6, but only up to about 100 °C. Figure 6b presents *E′* of the samples at temperatures of 30 °C, 60 °C and 120 °C. Compared to conventionally processed PA6, PA_LT exhibited higher *E′* at 30 °C (+10%), 60 °C (+17%) and 120 °C (+15%), which can be attributed to the supercooling of the melt in an injection moulding machine that resulted in higher molecular orientation, a higher degree of crystallinity, and lower free volume. The composite processed by a conventional two-step process (PA-C) exhibited a significant increase in *E′* compared to neat PA6, with increases of 41%, 57%, and 64% observed at temperatures of 30 °C, 60 °C, and 120 °C, respectively. Compared to the PA-C sample, the PA-C_LT sample exhibited slightly higher *E′* over the entire studied range, with increases of 4%, 6%, and 2% observed at temperatures of 30 °C, 60 °C, and 120 °C, respectively. The higher values of *E′* for PA-C_LT sample can be explained by the effects of LT processing on the matrix (orientation, crystallinity, free volume) and on CeF since a slightly higher orientation and lower degradation of the fibres can be expected due to the higher elongational flow and lower thermal stress. Composites processed in one step had up to 12% lower *E′* compared to composites processed in two steps. This can be explained by the lower amount of well-dispersed CeF, which was evident from macroscopic and microscopic observations as well as from DSC measurements (higher apparent degree of crystallinity). Among these composites, the PA-C_FDC sample had slightly higher *E′* values than the composites processed by the LT approach, which had *E′* at approximately the same level. Figure 6c presents tan *δ* of the samples at temperatures of 30 °C, 60 °C, and 120 °C. At 30 °C, the PA sample had the highest tan *δ* value (0.075). The sample PA_LT had a slightly lower tan *δ* value (0.071), which can be attributed to the effect of LT processing on the morphology of the matrix. Composites processed in two steps had lower tan *δ* values than neat PA6 at 30 °C, with a similar trend observed as for neat PA6, where LT processing resulted in slightly lower tan *δ* values. For composites processed in one step, tan *δ* at 30 °C was approximately at the same level (0.069–0.072), which was lower compared to composites processed in two steps. At 60 °C, the samples of neat PA6 had higher tan *δ* values (0.104 and 0.100 for PA and PA_LT, respectively) than composites, whose tan *δ* was more or less in the same range (0.090–0.093). In contrast, samples of neat PA6 had lower tan *δ* values than the composites at 120 °C. The reason for this behaviour could be due to the differences in the crystalline structure of the matrix between the neat PA6 and the composites. CeF hinders the crystallisation of PA6, which seems to result in slightly smaller crystals and a lower degree of crystallinity. In the glass transition region, the effect of the restriction of the molecular motion by the stiff CeF could be a larger contributor to the increase in elastic response, while at higher temperatures the effect of matrix morphology could play the predominant role. Figure 6d presents *T*_g_ and the corresponding tan *δ* peak values of the samples. The sample PA had a *T*_g_ of 57 °C and a tan *δ* value of 0.105, while the processing of neat PA6 at LT resulted in a slightly higher *T*_g_ (+1 K) and a lower tan *δ* peak (0.100). Composites processed in two steps had increased *T*_g_ and decreased tan *δ*. Composites processed in one step had a 1–3 K lower *T*_g_ compared to composites processed in two steps, which could be due to the higher moisture content of the composites due to increased absorption by the fibre agglomerates.

### 3.7. Tensile Properties

**Figure 7:**
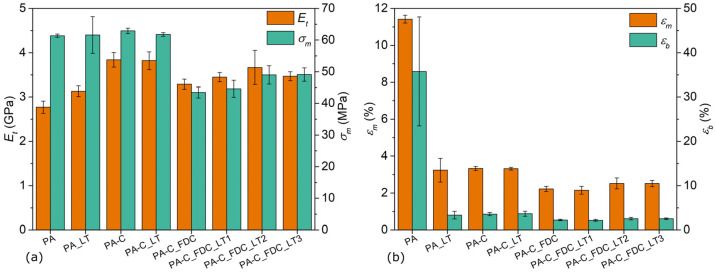
Tensile properties of samples: (**a**) modulus and strength; (**b**) strain at strength and strain at break.

The tensile properties of samples are presented in Figure 7. Neat PA6 processed by a conventional IM process had an *E*_t_ of 2.8 GPa, while PA6 processed by the LT approach had a slightly higher modulus (3.1 GPa). The average *σ_m_* of neat PA6 processed under both conditions was comparable (about 60 MPa), but sample PA_LT had a much higher deviation of the results. Large differences between the PA and PA_LT samples were observed in terms of strain at strength (*ε*_m_) and strain at break (*ε*_b_), which decreased from 11.4% to 3.2% and from 35.8% to 3.4%, respectively. While PA6 processed under conventional conditions was ductile, PA6 processed at LT was brittle but slightly stiffer. The former sample had a relatively high deviation of *ε*_b_ (±12.3%), which is a common phenomenon for non-filled PA6 [30,31,32]. Compared to neat PA6 (sample PA), the conventionally processed composites (PA-C) had a higher *E*_t_ value (+39%) and slightly higher *σ_m_* value (+3%). Similar to PA_LT, all composites had significantly reduced *ε*_m_ and *ε*_b_ and exhibited brittle fracture behaviour. Composites processed in two steps using an LT approach had slightly lower *σ_m_* (−2%) compared to conventionally processed composites, while other tensile properties did not differ significantly. In general, the samples prepared in one step had a lower *E*_t_ than those prepared in two steps. The sample PA-C_FDC_LT2 had the highest *E*_t_ value (3.7 GPa) among the composites processed in one step, which was not significantly lower than the *E_t_* value of the composites processed in two steps, but the measurement had a high standard deviation (±0.4 GPa). Other samples prepared by a one-step process had *E_t_* in the range of 3.3–3.5 GPa. In contrast, samples produced using a one-step process had significantly reduced *σ*_m_, ranging from 43 MPa to 49 MPa, depending on the processing conditions. The reduced *σ*_m_ clearly resulted from large agglomerates of non-dispersed fibres, which acted as stress concentration points that led to premature failure, which was also reflected in the high standard deviations of the measurements. The highest *σ*_m_ values (49 MPa) among composites processed in one step were observed for the PA-C_FDC_LT2 and PA-C_FDC_LT3 samples, which were processed with the LT approach and using higher *v*_screw_ and *P*_back_, which may result in slightly better dispersion of fibres and, in turn, better mechanical properties [33,34]. Similar trends to *σ*_m_ were observed for *ε*_m_ and *ε*_b_, both of which were lower for the composites processed in one step, while the PA-C_FDC_LT2 and PA-C_FDC_LT3 samples had slightly higher values of both quantities in comparison to other samples. While the composites exhibited increased *E*_t_, the *σ*_m_ was in a comparable range to neat PA6 or even lower for the composites processed in one step, despite fibres having much higher strength (*σ*_m_ of lyocell fibres ≈ 550 MPa [22,23]) than the matrix material. This is because the fibres used in the study were relatively short and poorly dispersed and oriented. It is well known that both *E*_t_ and *σ*_m_ (albeit to different degrees) of thermoplastic CeF composites are affected not only by *E*_t_ and *σ*_m_ of the matrix and fibres and fibre volume fraction, but also by their interface, fibre aspect ratio, and fibre clustering, which corresponds to available fibre stress transfer [1,35,36]. While LT-FDC resulted in significantly less degraded fibres, which theoretically should lead to better tensile properties of the composites, this was not the case in the present study due to poor fibre dispersion, which impedes effective stress transfer from the matrix to the fibre.

## 4. Conclusions

Composites based on PA6 and lyocell fibres were prepared using a novel LT-FDC approach, and their morphological, optical, thermal, and mechanical properties were compared with the composites prepared using a conventional two-step processing approach and with a neat matrix. Examination of the microstructure of the composites revealed that the composites prepared using LT-FDC exhibited poorly dispersed fibres compared to the conventionally processed composites. LT-FDC resulted in significantly less degraded CeF compared to the conventionally processed composites, which was characterised by higher L*a*b* values determined by colorimetry. The composites exhibited significantly higher *E′*, especially at higher temperatures, which is important for high-temperature applications. Although the composites processed with a conventional approach exhibited better mechanical performance than the composites processed with LT-FDC due to better fibre dispersion, the results are still encouraging for further development of the LT-FDC technology. The results showed that the LT-FDC processing approach resulted in significantly lower thermal stress on the sensitive CeF. While poor dispersion of the fibres was an issue in the present study, further developments of the process outlined in the paper may overcome this issue. In addition, a similar approach could be applied to IMC technologies based on twin-screw extruder design, which are known to result in much better fibre dispersion. The LT-FDC approach could also be applied to the preparation of other material combinations in which thermo-sensitive components are compounded into polymers. For some applications where the dispersion is not so challenging, the dispersive and distributive capabilities of the current system might be sufficient. The LT-FDC approach could offer not only technical advantages over conventional processing, but also environmental and economic ones, as two processing steps are combined into one.

## Figures and Tables

**Table 1:** Processing parameters for injection moulding and FDC.

Processing Parameter	Values and Units
Flat temperature profile	230 °C
LT profile	230 °C (nozzle), 210 °C (zone 7–12), 230 °C (zone 1–6)
Hot runner temperature	250 °C
Mould temperature	80 °C
Metering volume	500 cm^3^
Screw circumferential velocity (v_screw_)	15 m/min
Backpressure (*P*_back_)	3.5 MPa
Injection flow rate (*Q*_inj_)	100 cm^3^/s, 45 cm^3^/s (last 80 cm^3^)
Switch-over point	55 cm^3^
Packing pressure profile	60 MPa (0.5 s), 50 MPa (5 s), 40 MPa (2 s), 3.5 MPa (1 s)
Rest cooling time (*t*_cool_)	45 s

**Table 2:** Samples composition, processing techniques and processing parameters.

Sample	Material	Processing Technique	Processing Parameters Variation
PA	PA6	IM	/
PA_LT	PA6	LT-IM	/
PA-C	PA6/20% CeF	Compounding + IM	/
PA-C_LT	PA6/20% CeF	Compounding + LT-IM	/
PA-C_FDC	PA6/20% CeF	FDC	/
PA-C_FDC_LT1	PA6/20% CeF	LT-FDC	/
PA-C_FDC_LT2	PA6/20% CeF	LT-FDC	*v_s_*_crew_ = 30 m/min, *P*_back_ = 4 MPa
PA-C_FDC_LT2	PA6/20% CeF	LT-FDC	*Q*_inj_ = 200 cm^3^/s, *v_s_*_crew_ = 30 m/min, *P*_back_ = 4 MPa

**Table 3:** Thermal properties of samples determined by DSC.

Sample	*T*_c, onset_ (°C)	*T*_c_ (°C)	Δ*H*_c_ (J/g)	*T*_m_ (°C)	Δ*H*_m_ (J/g)	*X*_c_ (%)
PA	195.3 ± 0.2	192.2 ± 0.1	70.9 ± 0.5	220.8 ± 0.4	70.9 ± 0.5	30.8 ± 0.2
PA_LT	195.3 ± 0.1	192.3 ± 0.2	68.3 ± 5.7	221.3 ± 0.9	68.3 ± 5.7	29.7 ± 2.5
PA-C	193.9 ± 0.1	190.2 ± 0.1	54.4 ± 2.6	219.8 ± 0.3	54.4 ± 2.6	29.5 ± 1.4
PA-C_LT	194.0 ± 0.1	190.3 ± 0.0	54.1 ± 0.7	219.7 ± 0.4	54.1 ± 0.7	29.4 ± 0.4
PA-C_FDC	194.3 ± 0.2	191.0 ± 0.1	60.8 ± 1.6	220.2 ± 0.3	60.8 ± 1.6	33.0 ± 0.9
PA-C_FDC_LT1	194.5 ± 0.4	191.0 ± 0.5	59.8 ± 1.1	220.9 ± 0.7	59.8 ± 1.1	32.5 ± 0.6
PA-C_FDC_LT2	194.2 ± 0.4	190.7 ± 0.3	57.3 ± 1.4	219.7 ± 0.8	57.3 ± 1.3	31.1 ± 0.7
PA-C_FDC_LT2	194.5 ± 0.2	191.3 ± 0.2	58.6 ± 1.4	220.0 ± 0.2	58.6 ± 1.4	31.8 ± 0.8

## Data Availability

Not applicable.
